# Oral cancer awareness and screening practices in urban versus rural Indian population: Web-based survey

**DOI:** 10.6026/973206300211253

**Published:** 2025-05-31

**Authors:** Ipseeta Menon, Pratiksha Kumar, Pawan Rebello, Kaveri Surya Khanna, Robin Malik, Neha Agrawal

**Affiliations:** 1 Department of Public Health Dentistry, Kalinga Institute of Dental Sciences, KIIT University, Bhubaneswar, Patia, India; 2 Department of Oral Pathology and Microbiology, Government College of Dentistry, Indore, India; 3 Department of Oral and Maxillofacial Pathology and Oral Microbiology, Sardar Patel Postgraduate Institute of Dental and Medical, Sciences, Lucknow, India; 4 Department of Oral and Maxillofacial Pathology and Oral Microbiology, Yamuna Institute of Dental Sciences and Research, Gadholi, Yamunanagar, Haryana, India; 5 Department of Orthodontics and Dentofacial Orthopaedics, Santosh Dental College, Santosh (Deemed to be) University, Ghaziabad, Delhi NCR, India; 6 Department of Biochemistry, Index Medical College Hospital and Research center, Indore, India

**Keywords:** Oral cancer, screening, awareness, web-based survey

## Abstract

Cancer screening and awareness remain a vital strategy in the fight against cancer, helping to reduce both the incidence and mortality rates through early detection and intervention. Early detection significantly improves treatment outcomes and survival rates. Therefore, it is of interest to compare oral cancer awareness and screening practices among urban and rural Indian population, highlighting disparities, barriers and the need for targeted interventions to improve early detection and reduce mortality. Hence, a web-based survey was used to evaluate oral cancer awareness and screening practices among urban and rural populations. The analysis identified a significant disparity in awareness levels between the two populations, with urban respondents consistently exhibiting higher levels of awareness across all measured domains.

## Background:

Oral cancer includes cancers of the lip, other parts of the mouth and the oropharynx and combined ranks as the 13th most common cancer worldwide [1]. The global incidence of cancers of the lip and oral cavity is estimated to be 389,846 new cases and 188,438 deaths in 2022 [2]. It is a significant global health issue as its incidence, late-stage diagnosis and high mortality rates are increasing with each day. Despite being largely preventable and treatable when detected early, oral cancer continues to impose a substantial health and economic burden worldwide, particularly in low- and middle-income countries. A steadily increasing trend has been observed in the burden of oral cancer globally over the past few decades. From 1990 to 2021, the global age-standardized incidence rate increased from 3.26 to 5.34 per 100,000 and the mortality rate rose from 1.83 to 2.64 per 100,000 [3]. This rise is attributed not only to population growth and aging but also to the continued prevalence of significant risk factors. The World Health Organization (WHO) estimates that tobacco causes nearly 6.4 million deaths and hundreds of billions of dollars of economic damage worldwide each year [4]. Globally, an estimated 120 200 cases of oral cancer diagnosed in 2022 were attributable to smokeless tobacco or areca nut consumption, accounting for 30.8% of all oral cancer cases [5]. HPV-positive or opharyngeal cancers arising from the lingual and palatine tonsils are a distinct molecular-pathologic entity etiologically linked to infection by high-risk HPVs, especially HPV16 [6]. Therefore, it is of interest to compare oral cancer awareness and screening practices in urban versus rural Indian population using web-based survey.

## Materials and Methods:

A comparative cross-sectional questionnaire-based study was conducted over a period of three months among 700 participants from urban and rural settings. A structured questionnaire consisting of 13 questions was developed and a Google form link (Google form was generated in both English and Hindi with a separate link and proper mention of the language) was shared via WhatsApp group and shared with 700 patients. The questions aimed to assess participants' knowledge regarding the incidence, prevalence, etiology, treatment, prognosis, and clinical manifestations of oral cancer. All 700 distributed questionnaires were completed and returned, resulting in a 100% response rate. In urban areas to get an authentic picture of the result, participants were selected from different locations and professions without any socio-economic, gender, or geographical boundaries. Similarly, participants were selected in rural settings. The age group of participants selected was 15 years to 60 years. Informed consent was obtained through participation in the survey and participants were informed that participation is voluntary and on their responsibilities. The data was anonymous, no identifiable information was collected and no incentive was provided for completing the survey. To secure the confidentiality of study participants' data was stored in a Google sheet created for this study with limited access.

## Results:

The study evaluated oral cancer awareness among 700 participants, comprising 350 respondents each from urban and rural areas. The analysis revealed a marked disparity in awareness levels between the two populations, with urban respondents consistently demonstrating higher awareness across all assessed domains. Specifically, 90% of urban respondents had heard of oral cancer, compared to 65% of rural respondents. Awareness of key risk factors was notably higher in the urban group 85% recognized tobacco use as a risk factor and 80% identified alcohol use, versus 55% and 45%, respectively, in rural participants. Awareness of non-traditional risks-such as oral cancer in individuals who neither smoke nor drink was reported by 70% of urban and 40% of rural respondents. Exposure to educational materials, including posters and advertisements, was also higher in urban areas (75%) than in rural areas (35%). Regarding early detection, 80% of urban participants believed in its importance, while only 50% of rural participants shared this view. Similarly, 78% of urban respondents were aware of oral cancer symptoms, in contrast to 48% in the rural group. Discussions with healthcare professionals on oral cancer were reported by 60% of urban and only 30% of rural respondents. Knowledge of preventive measures also showed a gap: 85% of urban respondents knew that regular dental check-ups aid in early detection, compared to 55% in the rural group (Table 1). Lastly, 88% of urban respondents were aware that early diagnosis improves survival outcomes, while only 60% of rural participants recognized this fact. These findings highlight a significant urban-rural divide in oral cancer awareness, emphasizing the urgent need for targeted health education campaigns and improved outreach services in rural communities. In addition to awareness, the present study also assessed participants' access to and utilization of oral cancer screening services. The results revealed considerable differences between urban and rural respondents. Access to screening facilities was reported by 75% of urban participants, compared to only 40% in rural areas, indicating a significant gap in the availability or reach of healthcare infrastructure. Furthermore, the utilization of screening services was notably higher in urban areas, with 60% of urban respondents having undergone oral cancer screening, as opposed to only 25% of rural respondents. When asked about self-screening methods such as mouth self-examination, 65% of urban participants reported being aware of such practices, in contrast to a mere 20% of rural respondents (Table 2). These findings emphasize not only a disparity in awareness but also a serious inequality in access to care and proactive screening behavior, further underlining the necessity for community-based screening programs and health education initiatives in underserved rural regions.

## Discussion:

This study highlights a significant disparity in oral cancer awareness and screening practices between urban and rural populations. The findings indicate that urban respondents consistently demonstrated higher awareness levels across various parameters, including knowledge of risk factors, recognition of symptoms and engagement in preventive behaviors. The observed disparity between urban and rural areas is consistent with findings from previous studies. To illustrate, research conducted in Mandya, Karnataka reported that only 16.1% of rural participants had undergone oral cavity examinations in the past six months and a mere 7.7% had participated in oral cancer screening programs [7]. Similarly, a study from coastal villages in Tamil Nadu found that just 43.8% of participants were aware of oral cancer, with limited knowledge about its risk factors and symptoms [8]. Several factors contribute to this disparity. Rural areas often face challenges such as limited access to healthcare facilities, shortage of trained healthcare professionals and lower socioeconomic status, which collectively hinder the effective dissemination of health information and services [9]. This study found that individuals in rural areas possess limited knowledge about oral cancer's risk factors, symptoms, and the importance of early detection, which contributes to delayed diagnoses and poorer health outcomes [10]. Additionally, cultural beliefs and a lack of targeted health education programs further exacerbate the issue. To bridge this gap, it is imperative to implement community-based awareness campaigns tailored to the rural context. Utilizing local languages and culturally appropriate messaging can enhance the effectiveness of these interventions. Moreover, integrating oral cancer screening into existing primary healthcare services and training community health workers can improve early detection rates. Addressing the urban-rural disparities in oral cancer awareness and screening requires a multifaceted approach that combines education, infrastructure development and policy-level interventions. Such efforts are crucial for reducing the burden of oral cancer and improving health outcomes across diverse populations.

## Conclusion:

Cancer awareness in India remains low, especially in rural areas, due to disparities in education, access, and health literacy. Community-based, culturally tailored programs and integration of screening into primary care can enhance early detection. Sustained efforts and research into sociocultural influences are essential for long-term impact and equity in cancer care.

## Figures and Tables

**Table 1:** Response to oral cancer awareness among urban and rural populations

**Question**	**Urban Yes (%)**	**Rural Yes (%)**
Heard of oral cancer	90%	65%
Know tobacco is a risk factor	85%	55%
Know alcohol is a risk factor	80%	45%
Know non-users can also get oral cancer	70%	40%
Seen awareness posters or ads	75%	35%
Believe oral cancer can be detected early	80%	50%
Know the symptoms of oral cancer	78%	48%
Discussed oral cancer with a health professional	60%	30%
Know dental check-ups helps with early detection	85%	55%
Aware early diagnosis improves survival	88%	60%

**Table 2:** Response to screening questions among urban and rural populations

**Question**	**Urban Yes (%)**	**Rural Yes (%)**
Access to a nearby screening facility	75%	40%
Undergone oral cancer screening	60%	25%
Aware of self-screening methods	65%	20%
